# Functional response to cholinesterase inhibitor therapy in a naturalistic Alzheimer’s disease cohort

**DOI:** 10.1186/1471-2377-12-134

**Published:** 2012-11-05

**Authors:** Carina Wattmo, Åsa K Wallin, Lennart Minthon

**Affiliations:** 1 Clinical Memory Research Unit, Department of Clinical Sciences, Malmö, Lund University, Malmö, Sweden; 2 Memory Clinic, Skåne University Hospital, SE-205 02, Malmö, Sweden

**Keywords:** Alzheimer’s disease, Activities of daily living, Cholinesterase inhibitors, Treatment effect, Predictors, Statistical models

## Abstract

**Background:**

Activities of daily living (ADL) are an essential part of the diagnostic criteria for Alzheimer’s disease (AD). A decline in ADL affects independent living and has a strong negative impact on caregiver burden. Functional response to cholinesterase inhibitor (ChEI) treatment and factors that might influence this response in naturalistic AD patients need investigating. The aim of this study was to identify the socio-demographic and clinical factors that affect the functional response after 6 months of ChEI therapy.

**Methods:**

This prospective, non-randomised, multicentre study in a routine clinical setting included 784 AD patients treated with donepezil, rivastigmine or galantamine. At baseline and after 6 months of treatment, patients were assessed using several rating scales, including the Instrumental Activities of Daily Living (IADL) scale, Physical Self-Maintenance Scale (PSMS) and Mini-Mental State Examination (MMSE). Demographic and clinical characteristics were investigated at baseline. The functional response and the relationships of potential predictors were analysed using general linear models.

**Results:**

After 6 months of ChEI treatment, 49% and 74% of patients showed improvement/no change in IADL and in PSMS score, respectively. The improved/unchanged patients exhibited better cognitive status at baseline; regarding improved/unchanged PSMS, patients were younger and used fewer anti-depressants. A more positive functional response to ChEI was observed in younger individuals or among those having the interaction effect of better preserved cognition and lower ADL ability. Patients with fewer concomitant medications or those using NSAIDs/acetylsalicylic acid showed a better PSMS response.

**Conclusions:**

Critical characteristics that may influence the functional response to ChEI in AD were identified. Some predictors differed from those previously shown to affect cognitive response, e.g., lower cognitive ability and older age predicted better cognitive but worse functional response.

## Background

Functional ability is important in Alzheimer’s disease (AD), as it reflects the individual’s capacity to carry out daily activities (ADL) in real-life situations; thus, it may be indicative of how the patient is able to manage in society. Deterioration in daily functions accompanies the more commonly investigated cognitive symptoms in AD of impairment in memory, orientation and executive ability [1]. In addition to causing distress to the patient, a decline in ADL has a strong negative impact on the caregivers’ burden [2] and contributes to the rising societal cost of dementia [3]. A faster decline in function, rather than in cognition, has been described as a factor that could precipitate nursing-home placement [4,5]. A treatment that delays the deterioration of the individual’s functional abilities could possibly reduce the growing pressure on the family members and the usage of community-based care [3].

Currently, the main therapy used for mild-to-moderate AD is cholinesterase inhibitor (ChEI) treatment. ChEIs prevent the breaking down of acetylcholine by the acetylcholinesterase enzyme, resulting in increased levels in the synaptic cleft available for receptor absorption. This enhances cholinergic transmission and improves the communication between neurons [6]. Randomized AD trials have demonstrated that ChEIs are effective in slowing functional decline compared with placebo-treated controls [7-9], and “real world” studies have reported positive long-term effects of ChEI on ADL [10,11]. The expected effect of ChEI on function may slow or delay the deterioration rather than promote the regaining of lost abilities [12]. The clinical value of these drugs is still controversial among physicians. One study reported that the prevalence of ChEI treatment was lower among older than it was among younger AD patients [13].

Factors that might influence the short-term functional response to ChEI therapy in AD patients in naturalistic studies have not been investigated previously. In a recent paper, our group analysed the socio-demographic and clinical factors that might affect the long-term functional outcome in AD. A slower decline in ability to perform ADL after 3 years of ChEI therapy was associated with higher cognitive status at baseline, younger age, and the interaction effect of lower education and a longer time in the study. Moreover, a higher dose of ChEI, regardless of the drug used, or living with a family member predicted a better longitudinal outcome in IADL [10]. Some shorter naturalistic studies have observed different results in functional outcome between the three ChEI drugs [14,15].

The short-term cognitive response to ChEI therapy has been reported as heterogeneous among individuals with AD. Some studies have focused on different cognitive responses to ChEIs based on socio-demographic and clinical characteristics. A better cognitive response to treatment was observed in patients with a fast pre-treatment progression rate [16,17], in patients who were more cognitively impaired [9,18,19], in males [19,20], and in patients taking larger doses of ChEIs [19,21]; however, these results were not conclusive [22,23]. Inconsistent results have been described regarding age [14,24] and apolipoprotein E (APOE) genotype [9,25].

The identification of subgroups of functional responses to ChEI may provide valuable prognostic information to clinicians and social service providers and treatment recommendations for clinical practice, as well as increased knowledge regarding counselling patients and their families about the treatment effect.

The aim of this study was to identify the socio-demographic and clinical factors that have an impact on functional response after 6 months of ChEI therapy.

## Methods

### Study and subjects

The Swedish Alzheimer Treatment Study (SATS) was started to investigate the long-term effectiveness of ChEI treatment (donepezil, rivastigmine and galantamine) in naturalistic AD patients in a routine clinical setting. SATS is a 3-year, open-label, observational, non-randomised, multicentre study that was described in detail in a previous publication [26]. Most patients were in the mild-to-moderate stages of the disease and were prospectively recruited from 14 memory clinics in Sweden.

Seven hundred and eighty-four individuals with baseline Mini-Mental State Examination (MMSE) [27] scores ranging from 10 to 26 and a fulfilled 6-month post-baseline assessment were included up until the end of December 2005, thereby having the opportunity to complete the full 3-year SATS programme.

Outpatients aged 40 years and older who received the clinical diagnosis of dementia as defined by the Diagnostic and Statistical Manual of Mental Disorders, 4th edition (DSM-IV) [28] and possible or probable AD according to the criteria of the National Institute of Neurological and Communicative Disorders and Stroke and the Alzheimer’s Disease and Related Disorders Association (NINCDS–ADRDA) [29] were considered for inclusion. In addition, the selected patients had to be living at home at the time of diagnosis, had to have a responsible caregiver and had to be assessable using the MMSE at the start of ChEI treatment (baseline). Patients who did not fulfil the diagnostic criteria for AD or those already receiving active treatment with ChEI (i.e., the patients were naïve at baseline), or individuals with contraindications to ChEI were excluded from the study. If ChEI treatment was stopped or the patient switched to another ChEI agent or other dementia treatments such as memantine or study drugs were added, the patient was excluded from the study at that point. Medications other than anti-dementia drugs were allowed and documented during the study.

All patients and/or caregivers gave their informed consent to participate in the study, which was conducted according to the provisions of the Helsinki Declaration and was approved by the Ethics Committee of Lund University, Sweden.

The SATS patients were assessed in a structured follow-up program, which evaluated cognition, global performance, ADL and community-based service utilization at the start of ChEI treatment and semi-annually for a period of 3 years. The assessments at baseline were performed before and close to the start of ChEI therapy. The ChEI dose was recorded after 2 months of treatment, and every 6 months after the baseline assessment. Trained dementia nurses obtained the ADL evaluation from an interview with the caregiver. After inclusion and baseline assessments, the patients were prescribed ChEI according to the approved product recommendations. The choice of drug and dosage for each individual patient was left entirely to the physician’s discretion and professional judgment.

### Assessment scales

#### IADL

The Instrumental Activities of Daily Living scale (IADL) [30] consists of eight items: ability to use the telephone, shopping, food preparation, housekeeping, ability to do laundry, mode of transportation, responsibility for own medications and ability to handle finances. Each item was scored from 1 (no impairment) to 3–5 (severe impairment), giving a total range of 8–31 points. Some of the instrumental activities could be gender dependent among the elderly; therefore, a mathematical correction of the sum of the IADL scores was performed, to avoid these activities affecting the results. The transformation used the data from the rated items to estimate a total score within the range of the total IADL scale [5].

#### PSMS

The Physical Self-Maintenance Scale (PSMS) [30] consists of six items: toilet, feeding, dressing, grooming, physical ambulation and bathing. Each item was scored from 1 (no impairment) to 5 (severe impairment), allowing a total range of 6–30 points.

#### Cognitive tests

Cognitive ability was assessed using the MMSE, with a range from 0 to 30, in which a lower score indicates more impaired cognition, and using the Alzheimer’s Disease Assessment Scale-cognitive subscale (ADAS-cog) (0–70 points) [31], in which a lower score indicates better cognition.

#### Definition of functional response

The improved/unchanged groups were defined as ≥ 0 point change in IADL or PSMS score, respectively, between the start of ChEI therapy and the 6-month assessment. Consequently, the deteriorated group had lower ADL ability of at least 1 point after 6 months of treatment. To facilitate comparisons between the MMSE, ADAS-cog, IADL and PSMS assessment scales, changes in the scores reflected as positive values were interpreted as indicating improvement and negative values as indicating worsening.

### Statistical analyses

The Statistical Package for Social Sciences (SPSS) software (version 19.0; SPSS Inc., Chicago, IL, USA) was used to perform the statistical analyses. The level of significance was defined as *p* < 0.05 if not otherwise specified, and all tests were two-tailed. Parametric tests were used because of the large sample and the approximately normally distributed continuous potential predictors. Independent samples *t* tests were used to compare the differences between the means for the responder groups, and χ^2^ tests were computed for analyses of categorical variables. Pearson’s correlation coefficient was calculated to investigate any linear associations between continuous variables.

#### General linear models

In this study, we used general linear models to achieve a multivariate resolution in the analysis of the association between potential predictive independent characteristics, including a comparison of the three ChEI agents, on the functional response to treatment in a routine clinical setting. The dependent normally distributed variables were the change in IADL or PSMS score after 6 months, respectively. Several socio-demographic and clinical background variables were included in the models as independent variables. The selection of these variables was based on evidence-based knowledge and well-known risk factors of AD. The selected background variables were: age at first assessment, clinician’s estimate of age at onset or duration of AD, gender, years of education, APOE ε4 carrier status, solitary living, functional status at baseline measured by IADL or PSMS, cognitive severity at baseline measured by MMSE or ADAS-cog, medication use (antihypertensive/cardiac therapy, anti-diabetic drugs, lipid-lowering agents, estrogens, non-steroidal anti-inflammatory drugs (NSAIDs)/acetylsalicylic acid, anti-depressants, anti-psychotics or anxiolytics/sedatives/hypnotics), type of ChEI agent and drug dose.

Because of the strong linear correlation between MMSE and ADAS-cog scores, these variables were entered separately in the models. Similarly, the potential predictors of age at onset and duration of AD were also entered separately. The ChEI agents were coded as a set of dummy variables. The ChEI dose could vary during the treatment period for an individual patient and between patients. Therefore, the mean dose used during the first 6 months of therapy was calculated for each patient. Furthermore, to obtain a similar metric of percent maximum dosage for the three ChEI agents, the mean dose was divided by the maximum recommended dose for each drug agent, i.e., 10 mg donepezil, 12 mg rivastigmine and 24 mg galantamine. The rivastigmine dose refers to oral therapy. Finally, the possible interaction effects of ADL (IADL or PSMS) ability with gender, age or cognitive status were included in the models. The term “type of ChEI with dose” was also included. Non-significant variables (*p* > 0.05) were removed in a backward stepwise elimination manner. The hierarchical principle was observed in these analyses; terms that appeared in interactions were not considered for elimination.

## Results

### Responder groups

The 784 patients were divided into two groups according to the difference in IADL and PSMS score after 6 months of ChEI treatment: improved/unchanged (IADL, *n* = 383 (49%); PSMS, *n* = 578 (74%)) and deteriorated (IADL, *n* = 401 (51%); PSMS, *n* = 206 (26%)).

The socio-demographic and clinical characteristics of the two groups are displayed in Table 1. The improved/unchanged IADL group showed a significantly better cognitive status at baseline measured by ADAS-cog score (t(768) = 2.18; *p* = 0.030) (but not using the MMSE) and a lower instrumental ADL ability (t(782) = −2.82; *p* = 0.005). The improved/unchanged PSMS group was younger at the onset of AD (t(778) = 2.28; *p* = 0.023) and at the start of ChEI treatment (t(782) = 2.73; *p* = 0.007). This group also exhibited significantly better preservation compared with the deteriorated PSMS group regarding the mean MMSE (t(782) = −5.94; *p <* 0.001), ADAS-cog (t(768) = 6.53; *p <* 0.001) and IADL (t(782) = 7.15; *p <* 0.001) scores at the start of treatment. In addition, 23% of the patients in the improved/unchanged PSMS group used anti-depressants at baseline compared with 33% in the deteriorated PSMS group (χ^2^(1) = 7.45; *p* = 0.007).

**Table 1:** Demographic and clinical characteristics

	**IADL**			**PSMS**		
	**Improved/ unchanged**	**Deteriorated**	***P*value**	**Improved/ unchanged**	**Deteriorated**	***P*value**
**Variable**	*n* = 383 (49%)	*n* = 401 (51%)		*n* = 578 (74%)	*n* = 206 (26%)	
Female gender	232 (61%)	264 (66%)	0.138	360 (62%)	136 (66%)	0.356
APOE ε4 carrier, (*n*=771)	257 (68%)	268 (68%)	1.000	387 (68%)	138 (68%)	1.000
Solitary living at baseline	116 (30%)	141 (35%)	0.149	184 (32%)	73 (35%)	0.343
Antihypertensives/Cardiac therapy	156 (41%)	158 (39%)	0.716	237 (41%)	77 (38%)	0.407
Anti-diabetics	19 (5%)	14 (3%)	0.374	29 (5%)	4 (2%)	0.068
Lipid-lowering agents	46 (12%)	39 (10%)	0.358	65 (11%)	20 (10%)	0.603
Estrogens	21 (5%)	35 (9%)	0.095	37 (6%)	19 (9%)	0.206
NSAIDs/Acetylsalicylic acid	116 (30%)	117 (29%)	0.755	173 (30%)	60 (29%)	0.929
Anti-depressants	91 (24%)	107 (27%)	0.366	131 (23%)	67 (33%)	0.007
Anti-psychotics	11 (3%)	20 (5%)	0.145	20 (3%)	11 (5%)	0.296
Anxiolytics/Sedatives/Hypnotics	54 (14%)	51 (13%)	0.601	70 (12%)	35 (17%)	0.094
**Variable**	**Mean ± standard deviation**		***P*value**	**Mean ± standard deviation**		***P*value**
Estimated age at onset, years	71.8 ± 7.2	71.9 ± 7.7	0.969	71.5 ± 7.7	72.9 ± 6.8	0.023
Age at first assessment, years	74.8 ± 7.1	75.0 ± 7.1	0.678	74.6 ± 7.4	76.0 ± 6.3	0.007
Education, years	9.3 ± 2.4	9.4 ± 2.5	0.388	9.4 ± 2.5	9.3 ± 2.5	0.755
MMSE score at baseline	21.6 ± 3.8	21.1 ± 3.8	0.116	21.8 ± 3.5	20.0 ± 4.1	<0.001
ADAS-cog score (0–70) at baseline	20.0 ± 8.9	21.4 ± 8.9	0.030	19.5 ± 8.5	24.1 ± 9.1	<0.001
IADL score at baseline	16.4 ± 5.9	15.3 ± 4.8	0.005	15.1 ± 5.3	18.1 ± 5.1	<0.001
PSMS score at baseline	7.5 ± 2.2	7.5 ± 2.1	0.969	7.4 ± 2.1	7.7 ± 2.3	0.113
Number of medications at baseline	3.0 ± 2.5	2.7 ± 2.3	0.216	2.8 ± 2.4	3.1 ± 2.4	0.101

*ADAS-cog* Alzheimer’s Disease Assessment Scale-cognitive subscale, *APOE* apolipoprotein E, *IADL* Instrumental Activities of Daily Living scale, *MMSE* Mini-Mental State Examination, *NSAID* non-steroidal anti-inflammatory drugs, *PSMS* Physical Self-Maintenance Scale.

Older age at the start of ChEI therapy demonstrated weak linear associations with lower ADL (*n* = 784; IADL: *r* = 0.292, *p* < 0.001; PSMS: *r* = 0.249, *p* < 0.001) and cognitive abilities (ADAS-cog: *n* = 770, *r* = 0.098, *p* = 0.005), but not with measured MMSE score. A higher number of medications at baseline correlated weakly with increased functional impairment (*n* = 782; IADL: *r* = 0.170, *p* < 0.001; PSMS: *r* = 0.171, *p* < 0.001) and higher age (*n* = 782, *r* = 0.245, *p* < 0.001), but not with cognitive ability.

### ChEI treatment

The number of individuals (%) treated with donepezil, rivastigmine and galantamine was 436 (56%), 175 (22%) and 173 (22%), respectively. During the first 6 months of treatment, the mean ± standard deviation (SD) doses of donepezil, rivastigmine and galantamine were 6.2 ± 1.6, 4.9 ± 1.3 and 12.4 ± 3.1 mg, respectively. No difference in dose was detected between the improved/unchanged and deteriorated groups.

### Factors affecting the functional response to ChEI therapy

General linear models using change in IADL or PSMS scores after the first 6 months of treatment as continuous variables were built to identify the socio-demographic and clinical factors that affected the response in multivariate models. The degree of explanation of the variance in the models was moderate, IADL: (R^2^ = 0.310, *p* < 0.001) and PSMS: (R^2^ = 0.335, *p* < 0.001). The models and the significant predictors are presented in Table 2.

**Table 2:** Factors affecting the 6-month functional response to ChEI treatment (final general linear models)

**Dependent variable, change from baseline**	**IADL**			**PSMS**		
**Percentage of variance accounted for**	**R**^**2**^**= 0.310,*p*< 0.001**			**R**^**2**^**= 0.335,*p*< 0.001**		
**Significant predictors**	**β**	**95% CI (β)**	***p*value**	**β**	**95% CI (β)**	***p*value**
Intercept	−10.922	−15.585, –6.259	< 0.001	0.419	−2.156, 2.995	0.749
Age at first assessment, years	−0.041	−0.070, –0.011	0.007	−0.018	−0.036, –0.001	0.036
ADL score at baseline^a^	0.521	0.300, 0.741	< 0.001	−0.175	−0.441, 0.090	0.196
MMSE score at baseline	0.453	0.267, 0.640	< 0.001	−0.021	−0.128, 0.086	0.697
Number of medications at baseline			ns	−0.079	−0.134, –0.024	0.005
NSAIDs/Acetylsalicylic acid (no = 0, yes = 1)			ns	0.419	0.133, 0.705	0.004
*Interaction terms:*						
ADL baseline score^a^ × MMSE baseline score	−0.016	−0.026, –0.006	0.002	0.018	0.005, 0.032	0.008

Gender, APOE ε4 carrier status, solitary living, age at onset (or duration of AD), years of education and the variables comparing the ChEI agents and dose were not significant.

β values are expressed per 1 unit increase for continuous variables and for the condition present in dichotomous variables

^a^ADL = IADL or PSMS, respectively.

*ChEI* cholinesterase inhibitor, *CI* confidence interval, *IADL* Instrumental Activities of Daily Living scale, *MMSE* Mini-Mental State Examination, *NSAID* non-steroidal anti-inflammatory drugs, *ns* not significant, *PSMS* Physical Self-Maintenance Scale.

**Figure 1:**
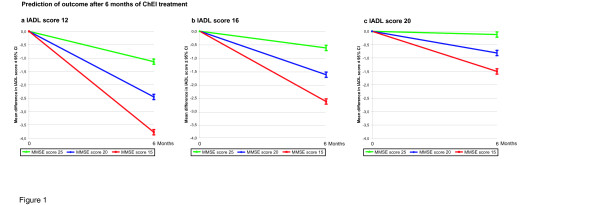
**Interaction effects between instrumental ADL and cognitive outcomes.** Six-month mean Instrumental Activities of Daily Living (IADL) scale outcomes with 95% confidence intervals predicted by the general linear models for patients with IADL scores of: **a.** 12, **b.** 16 and **c.** 20 at the start of ChEI treatment. A significant interaction effect was observed between cognitive and functional abilities at baseline (*p* = 0.002), i.e., a higher level of cognition and more impaired ADL ability implied increased response to ChEI therapy. In the figures, the calculated outcomes were based on a 75-year-old patient, and Mini-Mental State Examination (MMSE) scores (15, 20 and 25 were chosen as arbitrary examples) were used to illustrate the interaction.

**Figure 2:**
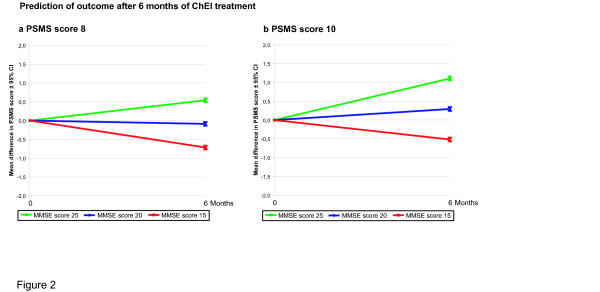
**Interaction effects between basic ADL and cognitive outcomes.** Six-month mean Physical Self-Maintenance Scale (PSMS) outcomes with 95% confidence intervals predicted by the general linear models for patients with PSMS scores of: **a.** 8 and **b.** 10 at the start of ChEI treatment. A significant interaction effect was observed between cognitive and functional abilities at baseline (*p* = 0.008), i.e., a higher level of cognition and more impaired ADL ability implied increased response to ChEI therapy. In the figures, Mini-Mental State Examination (MMSE) scores (15, 20 and 25 were chosen as arbitrary examples) were used to illustrate the interaction. The calculated outcomes were based on a 75-year-old patient who did not receive NSAID/acetylsalicylic acid treatment and had three medications at baseline.

Younger individuals showed a better response to treatment after 6 months regarding both instrumental and basic ADL abilities. In addition, there was a significant interaction effect between cognitive and functional abilities at baseline, i.e., a higher level of cognition and more impaired ADL ability implied increased functional response to ChEI therapy (Figures 1 and 2).

As an example, a subject with an IADL score of 12 and an MMSE score of 15 at baseline showed an additional 3.6 points of IADL deterioration after 6 months, on average, compared with an individual with an IADL score of 20 and an MMSE score of 25. The variable anti-depressant medications exhibited a trend towards significance in the IADL model (*p* = 0.058), indicating that depression may inhibit the response to ChEI therapy. Patients taking a higher number of medications at baseline exhibited a more negative treatment response, based on the PSMS score. In contrast, individuals treated with NSAIDs/acetylsalicylic acid at baseline showed a better response regarding basic ADL. Using ADAS-cog instead of MMSE score as an independent variable in the multivariate models yielded similar results; therefore, these results are not presented here.

The variables gender, carrier of the APOE ε4 allele, solitary living, medication use (antihypertensive/cardiac therapy, anti-diabetic drugs, lipid-lowering agents, estrogens, anti-psychotics or anxiolytics/sedatives/hypnotics), age at onset (or duration of AD), years of education, type of ChEI agent, drug dose and the interaction effects of gender × ADL ability, age × ADL ability or type of ChEI × dose were not significant when included in the models.

Better ADL ability at baseline and a more rapid functional decline after 6 months of ChEI therapy showed a weak linear relationship (*n* = 784; IADL: *r* = 0.198, *p* < 0.001; and PSMS: *r* = 0.140, *p* < 0.001). The initial change in MMSE score during the first 6 months of treatment exhibited a weak positive linear association with change in IADL (*n* = 778, *r* = 0.194, *p* < 0.001) and PSMS (*n* = 778, *r* = 0.130, *p* < 0.001) score, respectively.

### Functional outcome after 3 years of ChEI treatment

After 3 years, 339 patients (43%) remained in the study. No difference in completion rate was found between the improved/unchanged vs deteriorated IADL responder groups, but 47% of the individuals in the improved/unchanged PSMS group completed the study compared with 32% in the deteriorated group (χ^2^(1) = 14.91; *p* < 0.001). The difference in IADL score between 6 months and the baseline score exhibited a moderate linear relationship with the 3-year IADL difference (*n* = 309, *r* = 0.449, *p* < 0.001), whereas the 6-month difference in PSMS score showed a weak correlation with the 3-year PSMS difference (*n* = 309, *r* = 0.214, *p* < 0.001). The patients in the improved/unchanged IADL responder group after 6 months of ChEI therapy showed a significantly higher IADL status after 3 years than did the deteriorated group (mean ± SD, 20.0 ± 6.9 vs 22.5 ± 5.9 points; t(307) = 3.38; *p* = 0.001). The improved/unchanged PSMS responder group exhibited, on average, better 3-year basic ADL ability compared with the deteriorated group (9.5 ± 3.7 vs 11.8 ± 4.2 PSMS points; t(307) = 4.30; *p* < 0.001). The improved/unchanged responder groups received a greater percentage of the maximum recommended ChEI dose than did the deteriorated groups during the 3-year study: IADL: (68 ± 18 vs 65 ± 18 %; t(782) = −2.11; *p* = 0.035); PSMS: (67 ± 18 vs 63 ± 19 %; t(782) = −2.61; *p* = 0.009).

## Discussion

In this study, which was performed in a clinical practice setting, we found that 49% and 74% of the AD patients showed improvement/no change in IADL and in basic ADL ability, respectively, after 6 months of ChEI treatment. The improved/unchanged groups showed a significantly better cognitive status at baseline. Regarding improved/unchanged basic ADL, patients in this group were younger and used fewer anti-depressants. Moreover, there was a significant interaction effect between cognitive and functional abilities at baseline, i.e., better preserved cognition and more impaired ADL ability implied increased functional response to ChEI therapy. The outcome was similar regardless of the use of MMSE or ADAS-cog scores, which gives credibility to the results. Younger individuals also exhibited a better 6-month response to treatment. Patients taking a higher number of medications at baseline exhibited a more negative response in basic ADL, whereas individuals using NSAIDs/acetylsalicylic acids showed a better response to ChEI. No difference in ADL response was detected among ChEI agents after adjusting for background variables.

ADL performance in AD shows a moderate linear association with cognitive ability (*r* = 0.4–0.7) among both untreated [32,33] and treated patients [34]. Therefore, ADL scales are a complement and an essential part of the assessment of the patient and might produce a more complete understanding of the effect of AD therapies [35]. The pharmacological therapeutic drugs available currently, ChEIs, have yielded improvements, on average, in cognition in several 6-month randomised clinical trials [36] and have shown positive cognitive effects in longer-term naturalistic studies [26,37]. Conversely, ChEIs tend to provide a slowing or delay of functional decline, on group level, rather than an improvement over the first 6 months of treatment.

Patient characteristics that might affect the short-term effect of ChEI treatment on ADL ability have not been investigated in previous naturalistic AD studies. Our group has recently published a paper on the socio-demographic and clinical predictors that influence the long-term functional outcome in ChEI-treated AD patients [10]. The 6-month response of ADL demonstrated a weak-to-moderate linear relationship with functional status after 3 years. Thus, the factors that might predict the short-term ChEI response are not necessarily equal to those that predict the longitudinal outcome. Higher cognitive ability and younger age at baseline were both independent predictors of better functional short-term response to ChEI and long-term treatment effect. In the present study, fewer medications at baseline or NSAID/acetylsalicylic acid therapy predicted a more positive 6-month basic ADL response to ChEI; these variables were not addressed in our previous analyses of the 3-year functional outcome. A higher dose of ChEI and living with a family member were independent predictors of better long-term IADL ability, but not of the short-term response of IADL. The difference in the results of ChEI dose might reflect the regular dose titration that always takes place during the first weeks of treatment, which inevitably led to a lower mean dose of ChEI in the first 6 months. The current study shows that the patients in both the improved/unchanged IADL and PSMS groups after 6 months of ChEI therapy received a higher mean dose of ChEI during the following years than did the deteriorated groups and demonstrated better ADL status at the end of the 3-year study. This might imply that the individuals who responded and could tolerate higher doses exhibited a more positive long-term functional outcome. The faster IADL deterioration among solitary living AD patients observed over a longer period of time might reflect symptoms of depression, apathy and social isolation [10]. The heterogeneity in functional response to the initiation of ChEI therapy highlights the importance of identifying specific subgroups of patients with differential response to treatment. Rapidly progressing patients require more resources from caregivers and social services [33].

A significant interaction between cognitive and functional abilities at baseline regarding response to ChEI treatment was identified in this study of mild-to-moderate AD patients. Higher cognitive status and lower ADL ability implied increased functional response to ChEI therapy. Therefore, the effects of these domains should not be interpreted separately. The present study only demonstrated a weak linear association between cognitive and ADL response after 6 months; thus, the predictors of response might differ between the domains. Previous studies from our group [19] and from others [9,38], but not all [22], observed that more cognitive impaired individuals exhibited a better 6-month cognitive response to ChEI treatment, emphasizing the importance of not excluding this group from treatment opportunities.

Prior work has reported that cognitive deficits are a risk factor for functional impairment among the elderly, even after adjusting for socio-demographic and medical characteristics [39]. Bullock & Lane [40] reported improved executive function and attention among patients with dementia that responded to rivastigmine therapy. Moreover, a relationship between executive dysfunction and diminished instrumental and basic ADL was observed [41]. Better function in the above-mentioned cognitive abilities might lead to a more positive response in ADL. A recent 6-month study of donepezil [22] showed that the mild AD group (MMSE ≥ 21) exhibited significantly less decline in ADL compared with the moderate group (MMSE < 21), which agrees with our findings. In contrast, subsequent analyses of donepezil [42] and rivastigmine [38] clinical trials showed that the functional benefit of the drug compared with the placebo was greatest in the moderate and moderately severe AD groups. However, those studies did not address the cognitive-functional interaction effect or the multivariate impact of other background variables.

In the present paper, significantly fewer AD patients in the improved/unchanged basic ADL group used anti-depressants. This type of medication also tended to affect the IADL response negatively in the multivariate model (*p* = 0.058). Depression is considered as a characteristic of fronto-subcortical pathology. Patients with a significant burden of this pathology, concomitant with a heavier load of AD pathology, may show a more aggressive course of disease [40]. Furthermore, Atchison et al. [43] observed that the rapidly declining AD patients, as measured using basic ADL ability, had higher levels of self-reported depressive symptoms at the initial evaluation. Although significance was borderline in the current study, the findings indicate that depression, even if treated pharmaceutically, might be related to faster functional deterioration and less response to ChEI.

This study also demonstrated that individuals using NSAIDs/acetylsalicylic acid responded better to ChEI regarding basic, but not instrumental, ADL. A recent study from our group suggested that this category of medication was also a predictor of a more positive cognitive response to ChEI [19]. In contrast, a recent placebo-controlled randomised trial of NSAID treatment has failed to show any positive effects on cognition or function in AD [44]. One explanation might be the shorter treatment period in that clinical trial compared with the longer perspective of the naturalistic SATS patients. Furthermore, NSAID/acetylsalicylic acid therapy implies a symptomatic effect on pain, which in cognitively impaired elderly individuals may be more difficult to assess and recognize [45]. Under-treated pain can have adverse functional consequences and, therefore, analgesics might positively influence the response of ADL.

The predictors younger age or fewer concomitant medications (basic ADL only) independently demonstrated a better functional response to ChEI therapy in the present study; these individuals exhibited less ADL impairment at the start of treatment. In older patients, the functional response might be more influenced by physical disability, impaired senses or medical conditions not related to AD [46]. A linear association between cognitive status and number of medications at baseline was not observed in the present study. This agrees with another multivariate study from our group, in which concomitant medications were not a significant predictor of response regarding cognition [19]. In that study, older patients exhibited better 6-month cognitive response and long-term outcome. In contrast, older age predicted more rapid longitudinal decline in both instrumental and basic ADL abilities [10]. A more pronounced decline in daily function because of the natural aging process in older individuals, compared with cognition, might negatively affect the functional response observed in the present paper.

The advantages of the SATS are the 6-month, prospective, well-documented cognitive and functional evaluations after ChEI exposure in a large cohort of AD patients. The inclusion of everyday patients with co-morbidities and concomitant medications provides an essential supplement to data generated in clinical trials. The SATS, like other long-term naturalistic studies, is limited because it is not placebo controlled (because of ethical aspects) or randomised with respect to drug use. The treating physician, specialized in dementia disorders, decided the type of ChEI and the dose according to the standard routine in clinical practice. To minimize possible differences between the treatment groups, multivariate models that took into account demographic and baseline clinical factors were used. Other medical conditions, such as concomitant somatic diseases that may influence ADL ability, were not evaluated in the SATS programme. Therefore, the number of medications was used as an indicator of co-morbidity. The functional response to ChEI therapy might be complex among elderly AD patients in clinical practice. The inclusion of additional predictors, not assessed in the SATS, might influence the multivariate models and affect the individual’s outcome in ADL. However, the shorter follow-up time analysed in the present paper might imply less influence of factors associated with, e.g., multimorbidity in comparison with longer-term studies.

In the future, additional well-structured naturalistic studies will be required to advance our understanding of the significant predictors that independently modify responses to AD therapy [47]. The results presented here need to be confirmed by other studies using data from other naturalistic cohorts. The socio-demographic and clinical composition of the study population may be one of the explanations for the varying responses to ChEI observed in different AD clinical trials. A predictor such as age might provide different responses in different domains, and certain medications/co-morbidities, e.g., NSAIDs/acetylsalicylic acid and depression, might also alter the response [19]. The knowledge obtained from ChEI response in specific subgroups can increase the quality of care by aiding the clinicians and social services in decision making and planning for the future. Furthermore, it may provide more accurate information regarding treatment expectations in counselling to patients and families.

## Conclusions

In conclusion, critical characteristics that may influence the functional response to ChEI were identified in this naturalistic AD study. A significant interaction effect showed that higher cognitive and lower ADL abilities at baseline implied increased functional response to ChEI therapy. Younger age, NSAID/acetylsalicylic acid therapy and fewer concomitant medications also predicted a positive response. Some of these significant predictors differed from those observed earlier as affecting the cognitive response to ChEI, e.g., more cognitive impairment and older age predicted worse functional but better cognitive response. The patients in both IADL and PSMS improved/unchanged responder groups received a higher mean ChEI dose during the following years, indicating that they could tolerate higher doses. These individuals also exhibited better functional status, on average, at the end of the 3-year study. Thus, early identification of patients with a better probability of response is important to improve the effectiveness of ChEI therapy and possibly reduce the need and costs of care. Knowledge of differences in response is necessary to increase the understanding of ChEIs and to compile informed treatment recommendations for clinical practice. Functional ability is a key domain in maintaining independent living; therefore, functional assessments and response to therapy should be regarded as being at least as important as cognitive outcomes.

## Abbreviations

AD: Alzheimer’s disease; ADAS-cog: Alzheimer’s Disease Assessment Scale - cognitive subscale; ADL: Activities of Daily Living; APOE: Apolipoprotein E; ChEI: Cholinesterase inhibitors; CI: Confidence interval; IADL: Instrumental Activities of Daily Living Scale; MMSE: Mini-Mental State Examination; NSAIDs: Non-Steroidal Anti-Inflammatory Drugs; PSMS: The Physical Self-Maintenance Scale; SATS: Swedish Alzheimer Treatment Study; SD: Standard deviation.

## Competing interests

The authors declare that they have no competing interests.

## Authors' contributions

CW participated in the study, supervised the data collection, was responsible for the statistical design and for carrying out the statistical analyses, interpreted the results and drafted the paper. AKW participated in the study, assisted in analysing and interpreting the data and critically revised the manuscript. LM designed the study and critically revised the manuscript. All authors read and approved the final manuscript.

## Pre-publication history

The pre-publication history for this paper can be accessed here:

http://www.biomedcentral.com/1471-2377/12/134/prepub
